# “Saying No Without Saying No”: An Organizational Case Study on Assertive Communication Practices Among Nursing Workforce in Saudi Arabia

**DOI:** 10.1155/jonm/6671562

**Published:** 2025-10-26

**Authors:** Mansour Mansour, Sama S. Hammad, Abdelrahman Al-Anati, Lubna Khalid Alkhowiter

**Affiliations:** ^1^Nursing Department, Fatima College of Health Sciences, Ajman Campus, Ajman, UAE; ^2^Fundamentals of Nursing Department, College of Nursing, Imam Abdulrahman Bin Faisal University, 2835 King Faisal Road, Dammam 34212, Saudi Arabia; ^3^Nursing Department, College of Applied Medical Sciences-Jubail, Imam Abdulrahman Bin Faisal University, 2835 King Faisal Road, Dammam 34212, Saudi Arabia; ^4^School of Nursing, University of Wollongong, Building 41, Wollongong, New South Wales 2522, Australia; ^5^Pediatric Intensive Care Unit (PICU), Maternity and Children's Hospital, Al Imam Ali Ibn Abi Talib St, Al Muraikabat, Dammam 32253, Saudi Arabia

## Abstract

**Aim:**

To examine nurses and other healthcare professionals' perspectives on the sociocultural communication strategies that shape both expressing and responding to nursing assertive communication behaviors in Saudi Arabia.

**Background:**

Assertive communication skills are an integral part of safe nursing practice and are closely aligned with nursing empowerment. Saudi Arabia has unique sociocultural dimensions that shape how nurses express, respond, and adopt certain tactical choices when practicing assertive communication behavior with peers and other healthcare professionals.

**Methods:**

A descriptive, qualitative case study design focusing on one university teaching hospital in the Eastern Province of Saudi Arabia was conducted. Data were collected using four focus groups, four semistructured interviews with nurses, doctors, pharmacists, and senior executive hospital personnel, along with document analysis. Data collection is guided by the modified version of Garon (2012)'s three communication dimensions on speaking up and examines the sociocultural communication strategies within the contexts of assertiveness or saying “no” indirectly.

**Results:**

Analysis led to three major themes and seven subthemes: “Understanding the cultural context,” “Tactical choices for practicing assertiveness,” and “Change on the horizon.”

**Conclusion:**

This study provides a valuable perspective on how nurses and other healthcare professionals from Saudi Arabia view and contextualize assertive communication in their practice.

**Implications for Nursing Management:**

The findings highlight the importance of understanding communication strategies that are utilized by the nursing workforce from diverse working environments, with a sociocultural focus on assertiveness strategies that promote staff empowerment.

## 1. Background

Difficult communication and challenging social encounters dictate that healthcare professionals, including nurses, employ certain assertive communication skills to negotiate a satisfactory outcome. Assertive communication for individuals is referred to as the ability to make requests, actively disagree, express personal feelings, and initiate, maintain, and terminate conversations [1]. Within healthcare, assertiveness is a broader interpersonal communication skill where individuals express opinions or share knowledge with confidence, clarity, and respect for both one's own rights and those of others. Principles of assertiveness include self-awareness, emotional regulation, active listening, and boundary setting [2]. Evidence about advocacy communication from the literature suggests that strong advocacy not only enables two-way communication by expressing one's point of view but also openly questioning one's interaction to enhance mutual understanding [3].

Assertive communication and speaking up are two terms which continue to be used interchangeably in literature; however, speaking up against unsafe practice in the clinical setting is sometimes considered a type of assertive communication to challenge unsafe practice by questioning staff or simply exchanging views until a satisfactory resolution is reached [4]. Speaking up within healthcare is defined as using one's voice to express concern to another individual with power, addressing an identified concern [5]. A recent systematic review on assertive communication training for nurses reported that structured assertive communication training can improve nurses' speaking up behaviors in cases of medical errors [6]. Safety culture plays a critical role in shaping the healthcare professional behavior toward speaking up [7], and due to the complex interplay of many factors, which include leadership, teamwork, and communication style, it is difficult to predict which one mostly influences the assertive communication behavior [8]. This communication behavior may also vary across professions [9]. Many healthcare professionals, including nurses, do not communicate assertively when they need to do so. Nurses in particular are said to communicate passively, conforming to the stereotype of a “nice” nurse, and are less likely to provide constructive criticism to others [10]. Garon [11] found that assertive communication among nurses takes place within three dimensions. First, the organizational and individual influences such as the influence of peers, managers, cultural background, values, language, and educational background. Second, message transmission such as how safely a relevant message was delivered and received, and finally the outcomes, including what were the results of speaking up. Certain communication skills can help individuals to express assertive behaviors in a clear, yet respectful manner that avoids intimidating the person being challenged. These include maintaining eye contact, using clear and concise speech, speaking firmly and positively, being nonapologetic, and giving the same message verbally and nonverbally [12].

There is a growing body of research that highlights the role of sociocultural contexts in shaping the development and nurturing of assertive communication behavior in general and among healthcare professions in particular. The literature outlines two types of cultures that shape individual assertive behavior: Collectivist culture, where the individual prefers long-term relationships, values in-group harmony, and tends to avoid confrontation. In contrast, people from individualist cultures do not avoid confrontation since they prefer clarity of situation over harmony [13]. In the context of Saudi Arabia, there is a conspicuous respect for age and superiority [14], which makes practicing assertiveness more challenging to fulfill, even for those who are skillful communicators in their work and social settings. The Saudi culture firmly subscribes to the collectivist end, where people prioritize cultural, social, and familial responsibilities [15]. This might be attributed to the fact that the status of individuals in Saudi Arabia is derived from their memberships of the group family, village, and tribe and is not usually determined by their individual capacities. Consequently, the entire family feels ashamed and equally responsible when one member is engaged, for example, in a dishonorable act. A study conducted by Alghamdi and Alqarni [16] found that Saudi participants rarely said direct no in their responses (only 5% of them), compared to the Americans who said “no” more frequently. This was due to the Saudi inclination to avoid a direct “no,” which is ascribed to the collectivistic Saudi culture, where harmony among group members is of utmost importance.

In standing up for themselves, individuals were reported to adopt tactical choices that would accommodate the local sociocultural requirements which would best convey the intended message in a clear, strong, yet culturally-endorsed manner. For example, in Asian cultures, a significant priority is assigned to harmony within the group, respect for those with more authority, and the importance of saving face (i.e., avoiding humiliation and embarrassment) in social relations [17]. While this could be true for other cultures, it is of particular significance in Asian cultures, which dictate using specific socially-acceptable procedures to avoid emotional bursts when exercising assertiveness with other people. In doing so, certain tactical communications strategies are likely to emerge in efforts to accommodate both expectations. Nursing empowerment is closely aligned with assertive communication among nurses and the wider healthcare workforce [18], and to examine what shapes the assertive communication attitudes among Saudi nurses, current and future research must capture those communication strategies and tactical choices that are endorsed by Saudi culture when practicing assertive communication behavior among nurses, fellow peers, and other healthcare professionals.

## 2. Objectives

To examine nurses' and other healthcare professionals' perspectives on the sociocultural communication strategies which shape both expressing and responding to nursing assertive communication behaviors in Saudi Arabia.

## 3. Methods

### 3.1. Theoretical Framework

**Figure 1:**
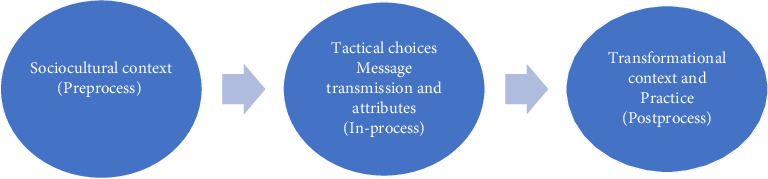
Theoretical framework (modified version of Garon [11]).

The study adopted a modified version of Garon (2012)'s three communication dimensions on speaking up among registered nurses. The model divides the process of speaking up among nurses into three stages: Influences on speaking up, message transmission and reception, and outcomes or results. In the current study, a modified version of this model was adopted not only to better fit the Saudi cultural and healthcare context but also to accommodate critical issues which are distinctively relevant to Saudi culture. The proposed framework is guided by three domains as follows: Cultural context influencing practicing assertive communication (premessage); tactical choices for transmitting, receiving, and practicing assertive communication (message attributes); and transformational practice (postmessage) (Figure 1). The theoretical framework emphasizes the importance that managers set an open communication culture [11]. When focusing on the antecedents of the framework, and for assertive communication to thrive, certain antecedents need to be present for nurses' experiences. First, organizational culture, where, for example, nurses must be encouraged to speak up during safety and multidisciplinary rounds despite cultural differences. Second, leadership support, where nurse managers listen actively, validate concerns, and respectfully challenge unsafe practices; they are in hand modeling assertive behavior and thus, empower staff nurses to model similar behavior. Third, training and education, where nurses are engaged in assertiveness training that can incorporate role-playing on how to address a colleague about a missed allergy documentation and thus builds confidence during training [19–21].

### 3.2. Design

The study utilized a descriptive, qualitative single case study research methodology, where the details of how nurses express and respond to assertive communication phenomena are described, when it is practiced within its social setting [22]. Descriptive case studies attempt to fully describe the different attributes of a phenomenon in its context, which can then be used for theory building. Three qualitative data collection methods were employed in this study: Focus group interviews, semistructured interviews, and document analysis. The qualitative method is best suited to explore participant's views on the cultural dimensions of communication attributes that are under investigation [23]. A complex interplay of several factors shapes the sociocultural context of assertive communication culture in Saudi Arabia. Qualitative research is said to be the most appropriate for studying the cultural dimensions of phenomena [24]. Using multiple data collection methods in this study helps to tackle assertive communication behavior from different perspectives, thus enriching the quality of data sought from the participants [25].

### 3.3. Participants and Settings

Data collection for this study took place in a 440-bed teaching hospital in the Eastern Province of Saudi Arabia. Registered nurses from diverse managerial levels, medical doctors, and clinical pharmacists, as well as senior hospital management staff, were invited to participate in this study. Registered nurses are likely to provide expert views on communication attributes from both ends of the continuum (as someone who challenges or responds to being challenged). It was anticipated that doctors' and pharmacists' views would shed light on their experience with nursing staff in terms of exhibiting and responding to assertive communication practice.

To be eligible to participate in the study, the participant must be working as a full-time state-registered nurse, doctor, or pharmacist and have been working at the hospital for at least 2 years and have had a direct clinical or managerial responsibility. Those who have been working in the hospital for less than 2 years were excluded (even if they were in senior positions). This is to make sure that the participants have solid insight into the communication and working culture in the hospital, which would allow them to reflect more thoroughly on their experience.

### 3.4. Data Collection

#### 3.4.1. Focus Groups

It is anticipated that rich qualitative data sought from the focus group discussion will not only provide valuable insights into the participants' views on the chief but also subtle, verbal, and nonverbal assertive communication patterns that are socially acceptable and culturally endorsed in Saudi Arabia. Four focus group interviews were conducted as follows: Three with nursing staff (Saudi front-line nurses, non-Saudi front-line nurses, and middle management nurses such as nursing supervisors), and one focus group with other healthcare professionals (doctors and pharmacists). Each group consisted of five to seven participants. Employing multiple focus groups promotes the examination of commonalities and differences in how health professionals' focus groups discussed the perceived communication styles as well as the sociocultural aspects that underpin effective assertive communication skills, albeit from each group's perspective.

#### 3.4.2. Semistructured Interviews

Semistructured interviews were conducted with key study informants in the hospital setting. This included the chief nurse at the hospital and assistant chief nurse, medical director, and director of the pharmacy department. The views of the key informants provided unique perspectives on the managerial and institutional approaches for developing, responding to, and nurturing assertive communication skills among the nursing workforce.

### 3.5. Document Analysis

Document analysis is a systematic evaluation of documents to provide a rich description of a single phenomenon [25]. It is often utilized to complement data collected by other methods, thus improving the credibility of the research findings [26]. Document analysis was used in this study to examine the hospital's written documents in relation to developing and practicing assertive communication styles among hospital nurses. The selection of these documents was based on their importance, reliability, relevance to the topic of assertive communication, and those that are readily accessible in the public domain or accessing them poses no ethical or professional risk. These documents included the following: Skills manual, teaching material, work policy, protocols and procedures, and event programs (i.e., printed outlines); letters and memoranda; newsletter, press releases, and organizational reports; communication policies, readily accessible internal memos, work regulations, training competencies portfolio, in-house face to face and online training material, hospital announcement, sources available on the hospital intranet webpage, relevant accreditation documents, professional training portfolios, and any published research data from the hospital setting. Around 40 documents were collated and analyzed, and some of these documents were large and exceeded 50 pages (i.e., training portfolio), and others were simply one page (i.e., internal memos). The majority of these documents were accessed directly by the research team, as it was available in the public domain, and only a few of them needed permission from the hospital to gain full access (i.e., training portfolio and internal police). The review of these documents not only contributed to exploring how far the knowledge and regulation of assertive communication behaviors were embedded, practiced, and followed up but also examined how the local sociocultural practice of assertive communication skills is shaping the written evidence on the hospital level.

### 3.6. Sample Size, Sampling Technique, and Recruitment

To recruit the participants, a purposeful sampling technique was used [27]. An email invitation was sent to all registered nurses, doctors, and pharmacists via a third party, who had legitimate access to the names and email accounts of the potential participants in the hospital where this study was carried out. If the recruitment of the participants is proven to be difficult using this recruitment technique, then a snowballing sampling approach will be used to recruit more participants, where those who agreed to participate in this study are asked to invite colleagues who meet the inclusion and exclusion criteria to participate in this study.

The number of focus groups and participants was initially guided by previous studies with a similar context, which suggested that four focus group interviews with participants from clinical settings can be sufficient [11], although the final number of focus groups was decided based on the data saturation in the interviews [28]. Four semistructured interviews with key informants (chief nursing director, assistant director of nursing for education and professional development, medical director, and director of the pharmacy department in the hospital) were also conducted in this study. For the document analysis stage, Bowen [25] emphasized that the quality of documents, rather than how many documents, should guide how far the document analysis should go. For this reason, depending on the quality of the documents that can be accessed, the research team sought to use a purposeful and systematic approach for selecting these textual sources, with an iterative, stepwise process of selection to the point of saturation [27].

### 3.7. Data Analysis

The focus group and semistructured interviews were digitally recorded with the consent of all participants. The data were then transcribed verbatim. The interviews were conducted in English, which is used as the medium of communication among healthcare professionals in the hospital where this study took place. The data were then analyzed using Clarke and Braun's [29] six-step thematic analysis. The thematic analysis follows a structured, sequential approach to interpreting research data and what distinguishes it from other qualitative data analysis approaches, such as grounded theory; it allows sufficient flexibility to capture naturally occurring data and themes to emerge, leading to a wide range of applications [29]. For example, thematic analysis allows for both inductive and deductive data analysis [30]. Such a systematic approach to data analysis consolidates consistency and replicability of the findings and captures the connections between the data, interpretation, and conclusions [31].

Two members of the research team conducted the initial data analysis independently, and then they met up to compare the extracted themes and agreed on the final data coding. Any discrepancies were referred to a third researcher, who analyzed the specific themes, and collectively, all researchers involved agreed on the final coding blocks. Similarly, thematic analysis was also utilized to analyze data collected from the documentary analysis stage, although findings from this data collection method were outlined in relation to those themes that emerged from the focus group and semistructured interviews.

### 3.8. Data Rigor

To ensure the credibility of data analysis, two researchers initially analyzed the data independently, and they then met up to compare and confirm their analyzed codes and agreed on generic themes. Any views that are not consistent with the overall trend (i.e., negative cases) have also been pursued and analyzed.

The transferability of the data was ensured by providing a “thick description” of the study settings, participants, and other methodological aspects. This is to ensure the conceptual generalization of the findings [32]. The study's findings are likely to be transferable to other healthcare settings within the Gulf Cooperation Council (GCC) countries regions which share a similar sociocultural context as Saudi Arabia. The hospital where this study took place shares a great deal of similarities with other hospitals operated by the Ministry of Health in Saudi Arabia, which further facilitates the transferability of the findings.

Being reflexive is an important skill that must be practiced in qualitative inquiry, both in data collection and analysis [33]. Although all research team are nurses by background, some of them are Saudi nationals and are likely to have some prior, potentially similar experience to the participants. Being reflexive meant those researchers are bound to make conscious attempts to avoid the risk of overfamiliarization with the settings or the participants' experiences, which might lead to the researcher making assumptions without seeking the rationale underpinning particular actions.

It is imperative to remain inductive during data analysis, and different strategies were employed to pursue this approach. For example, researchers have frequently documented their thoughts and decisions and discussed them with other researchers in the team to safeguard against becoming deductive and to ensure that their thinking has not become too much driven by the theoretical framework of the study [34].

### 3.9. Ethical Considerations

A participant information sheet (PIS) was emailed to all potential participants and was handed over again to the participants to read before the start of the focus groups and the semistructured interviews. The PIS explained the purpose of the study, the likely risks and benefits, and what the participants had to do if they wanted to participate. It stressed the voluntary nature of participating, and the fact that the participants can withdraw at any time without giving any reason. Each participant signed a consent form before the start of the interviews. All participants are invited to express their views without necessarily linking them to their personal experience, thus allowing a more supportive and less threatening approach to recruiting the participants. Full approval from the relevant institute review board (IRB) was secured before the start of data collection.

## 4. Results

Four focus group interviews (with 24 participants) were conducted with nurses, doctors, and pharmacists from different professional ranks, in addition to four semistructured interviews with senior-level managers across medicine, pharmacy, and nursing disciplines (Table 1).

Three overarching themes emerged from the data analysis relating to practicing assertive communication skills among the participating nurses: Understanding the cultural context, tactical choices for practising assertiveness, and change in the horizon. The three themes are discussed along with their relevant subthemes as presented in Table 2.

### 4.1. Understanding the Cultural Context of Assertiveness

This section illustrates the participant's views on how assertive communication is contextualized, understood, and practiced among healthcare professionals, including nurses, taking into consideration the Saudi cultural context. This illustration is critical to set the scene for the reader and provide foundational information which helps to better understand the contributions of the subsequent themes and subthemes in shaping assertive communication practice among nurses and other healthcare professionals involved in this research. Three subthemes are outlined here as follows: Swimming against the current, being acquainted with doctors and nurses, and the context of expat nurses.

#### 4.1.1. Swimming Against the Current

The participants' views, from all backgrounds, suggest that there are expectations within the working context to submit complete obedience to those in power, and there is an expectation that subordinate staff are not expected to challenge the decisions of more senior ones. One participant portrayed how any attempt to challenge the decisions of senior staff can be regarded as swimming against the current:

…most of the people think … (that) if I am your superior, you have to be obedient … the culture doesn't expect that you are doing a move … swimming against the current. Male clinical pharmacist

This practice seems to represent a key subset of the professional identity of such working culture, not only within the nursing profession but also when those being challenged come from a profession which is (rightly or wrongly) perceived to be in a higher professional hierarchy. For example, a nurse described how a doctor's order must be followed even though she had some concerns about the safety of the decision-making process.

Because he (doctor) was forcing us to do something that was against the policy, and then I was explaining it to him then “Doctor, we can't do that because it will be a problem to us if we will follow you even though you are a senior even (he) will say that “Oh it's okay” … because doctor is so high in a higher position, the admin will understand he was…” No, don't worry. Just go with that procedure. I will answer any problems after”. Female non-Saudi nurse

In this context, any nursing staff who does not subscribe to this practice and continues to challenge other senior members' decisions at work is seen by the wider team members as outspoken, and his or her behavior is perceived as crossing the boundaries and the established red lines.

Yes … yes…they (medical doctors) see her (the assertive nurse) as outspoken… Female medical doctor

Subsequently, the nurses' assertive views are to be devalued, and not taken seriously.

But still, in our (working) culture, an assertive nurse (views) is not valued… Female clinical pharmacist

Although a female medical doctor agreed with the existence of such an obedience culture in the hospital working environment, she was more cautious in generalizing this claim to all workers, including nurses.

No, no … you cannot generalize that everyone who works in the hospital is in complete obedience. Female medical doctor

#### 4.1.2. Being Acquainted With Doctors and Nurses

The participants' views suggest that there is an implicit work expectation that getting to know the doctors and nurses will make the task of challenging their work-related decisions less problematic. This works particularly well for those senior nurses who are perceived to have competent clinical skills, where their “assertive views” are welcomed and given more latitude by other healthcare professionals.

I experienced this after like one year, two years, three years now, when we become charge nurses, um, they (the doctors) know us. Everybody knows us, even the consultants know it's easy and more than … before… to talk with them, to guide them regarding any issue. Saudi male nurse

Another nurse recalled a situation where he had to challenge the decision of a medical doctor over the phone. The doctor did not initially recognize the identity of the nurse, and there was a moment of inevitable confrontation, but when the doctor recognized the name of the nurse, this helped to diffuse the tension, and the situation was quickly de-escalated.

… at first, he (medical doctor) wasn't able to recognize me but when he recognized me he was like, wait, uh, we are colleagues for a long time, this (confrontation) should not happen. Saudi male nurse

Similarly, both doctors and pharmacists appear to be mindful of the importance of having a good and comfortable working relationship with the nursing staff. In their views, this would help to facilitate their daily work in the wards. To keep this positive relationship, the nursing staff must be allowed a degree of flexibility where they, the nursing staff, are allowed, and indeed welcomed, to challenge the decision of other healthcare professionals, namely the doctors and the pharmacists.

It's (she-the nurse) is going to give you all the information. So you have to approach her in a nice way and not approach her while she is busy. And you have to find ways on how to be nice for them. And if you're nice, they're nice (to you). Female clinical pharmacist

*… If* we give an order or anything, it's always the nurse. I have to be nice to the nurse. But then in the clinic, we have certain nurses, we know them. Female doctor

On the other hand, being a new nurse who is unfamiliar with other members of the healthcare professionals may sometimes lead to wrongly articulating the different professional status attached to the other team members, such as doctors, and until the nurse becomes personally acquainted with peers and other team members, he/she is bound to receive a defensive backlash, therefore exercising certain communication skills, such as being assertive, can be temporarily problematic for those nurses.

#### 4.1.3. The Context of Expat Nurse

The views of expat nurses frequently illustrated how their sense of job insecurity and their perception of being outsiders to the Saudi culture have led them to keep a low profile and become reluctant to exercise assertive communication, particularly with Saudi medical doctors who are perceived to be in a higher professional status. For many expat nurses, exhibiting assertive behavior was synonymous with directly confronting those Saudi nursing peers and Saudi medical doctors, something they often strived to avoid.

… sometimes the (expat) nurse will be afraid… to lose her job. or they will complain against this nurse, she is afraid she will not ……… Like what she says, so these staff (Saudi) she can have an authority. She reported to her manager, she will speak up but, that one (expat nurse) she would keep quiet and she sometimes you don't want to… Female Saudi supervisor 3

Rather than opting to do the assertive behavior themselves, which may come with costly consequences, and to avoid any emotional and psychological outbursts that come with any direct confrontation with other healthcare professionals, the expat nurses seem to adopt a less sophisticated strategy by simply letting other Saudi peers do the “assertive” work on their behalf. For them, the Saudi nurses are in a better position to challenge their Saudi peers and Saudi medical doctors, and to diffuse any potential confrontation because they, the Saudi nurses, share the same cultural attributes, values, and expectations with those being challenged:

…when they (Saudi medical doctor) came to the unit, that time they were shouting and in the phone … So angry, and then when they spoke to this Saudi nurse, the doctor was just laughing… it seems like there is no issues. I don't know if this maybe because of the culture … it's easy for them to understand each other because of course … it is their country and for us, expats, you know … Female non-Saudi nurse

Some expat nurses stated that they would have a hard time working in the evening shift because, compared to the morning shift, there is likely to be a smaller number of Saudi nurses who can embark on such challenging social interaction (i.e., being assertive) with other Saudi nurses and doctors:

The Saudi (nurses) will be there in the morning shift. So the morning shift is OK. Mostly the worst time for us is the evening time, with less [Saudi] staff, so that time is more problem …You've got somebody (in the morning) with you to talk. Female non-Saudi nurse

This assumption was echoed by a Saudi nurse who reiterated that being and working in their home country and belonging to the same inner cultural circle of Saudi peers and doctors meant that Saudi nurses are more able to express assertive communication with those who share the same cultural backgrounds.

Because you are Saudi, it's your place because they (expat nurses) are coming from the other country. They feel this is not their home, for us we feel this is like our house. Saudi male nurse

### 4.2. Tactical Choices for Practising Assertiveness

This section explains the tactical approaches that the nurses were utilizing in practising assertive communication skills, and how their peers and other healthcare professionals are responding to these tactical choices.

#### 4.2.1. Avoiding Public Showdown

There was an almost unanimous agreement among the participants that publicly challenging other healthcare professionals is seen as provocative, insulting, and attacking the integrity of the person being challenged. The magnitude of this feeling appears to be profession-bound. For example, it is more visible when the nurse is publicly challenging a medical doctor (compared to fellow nurses) in front of other nurses, doctors, patients, and family, as one doctor put it:

Yes. Because you (as a doctor) would feel that she (the nurse) questioned me in front of people. What would they (other healthcare professionals) think of me? Uh, or she is challenging me exactly in front of people … Embarrassing, embarrassing… embarrassing… Female doctor

One nurse acknowledged that when he tried to challenge a doctor during a clinical procedure, the doctor's response suggested he (the medical doctor) felt devalued.

It is like you are devaluing him … for example, I experienced this with one doctor when he was doing a procedure. Female non-Saudi nurse

One nursing supervisor expressed a more explicit stance and suggested that simply raising the voice tone in front of the public can be considered insulting to the other person.

…tonewise, they (nurses) should not raise their voice … not at all. Female Saudi supervisor

To avoid such perceived public humiliation associated with exerting assertive communication, any nursing attempt to practice assertively can be accepted if it is first done privately and then off the public domain. This tactic seems to be culturally acceptable because it safeguards the personal and public image of the individuals being challenged, hence, making them more likely to respond more constructively. One pharmacist described how he would employ this tactic with nurses, doctors, and fellow pharmacists.

…if it (challenging others practice) involves public, they (nurses, pharmacists, and doctors) will consider it as a challenge, So, I try to take them aside and tell them. And I tried, uh, many times if I tell them in public, they will consider it a personal challenge and they refused. If I take them a side, they would accept. Male clinical pharmacist

In addition to having the conversation in private, one senior nursing staff also suggested that there are other situational prerequisites for accepting being challenged by nurses, including voice tone, eye contact, and facial expression.

I think … it (accepting being challenged) depends in the tone of voice, the body posture the facial expression how they are contacting each other by eye contact… Assistant director of nursing

#### 4.2.2. Aiming for a Compromise

This subtheme discusses how some nurses have adopted tactics to convey assertive communication behavior in a way that is tailored to the prevailing work and cultural contexts. In doing so, their main strategy was to accommodate both disagreement with the decision of those being challenged but equally avoiding direct confrontation with them so as not to provoke the cultural discourse which does not explicitly endorse direct clash with those in power.

One tactic which seems to be popular is acquiring the knowledge and expertise related to clinical settings, hospital policies, guidelines, clinical procedures, and clinical practice. This seemingly powerful asset will earn the nurse respect and appreciation among his/her peers and other healthcare professionals. This respect is then translated into practice by implicitly granting the nurse the right to challenge any decisions, and others (who are being challenged) would compromise and accept counter opinions expressed by nursing staff because, in their views, it is coming from someone with established expertise whom they know and trust.

For some doctors, when they are doing a procedure, and they feel that nurses are more experienced with this procedure or know the patient, they will ask also what do you think we should do here…for example, they are doing the procedure you are familiar with some and that's okay… they will accept (your views). Male non-Saudi nurse

Those senior nurses such as charge nurses or nursing supervisors are particularly viewed by many team members (i.e., doctors and pharmacists) as experts in their field, and therefore, their assertive attitudes are accepted and taken on board. One nurse compared how his views were considered when he was a junior nurse, and after he was promoted to a senior role.

…because they will ask the senior…will ask the charge nurse. Even if you are the one who is assigned to the patients, I'm talking about the doctors and maybe some other healthcare providers also… I experienced this after like one year, then after two years, three years now, when we become charge nurses, they know us. Everybody knows us, even the consultants now it's (challenging them) easy and more than before to talk with them, to guide them regarding any issue. Male Saudi nurse

Rather than directly exercising assertive sentences, several nurses have adopted commonly used tactics by reframing the assertive narrative into a question-like message and throwing a simple question in the hope that the doctor will pick up the underlying clues and revise his/her decision. This seems to be a particularly acceptable practice across the board for nurses, doctors, and pharmacists:

If they (nurses) have questions or they're not sure of one of the patients or the treatment or the dose, they would come and be nice. They will ask if the dose… this dose is right? Can you please see this? Can you read it again? Can you see the lab results? They're not aggressive. They would ask and they always put it in a question: Doctor: are you sure you have this? Do we document this as the medication dose? Female doctor

I will say like, doctor, have you noticed that? Or maybe you did notice that this patient was on contact precaution? Maybe you have to wear PPE, as per the policy. Male Saudi nurse

Even the pharmacist would employ the same tactic to convey implicit disagreement with the doctor but, in a question-like style.

…if the medication, for example, it's not the proper medication, I'm just trying to say … to show it as a question: Isn't this drug better than this one? Male clinical pharmacist

### 4.3. Change in the Horizon

The third theme highlights the participants' perspectives on the impact of current and forecasted work and role changes on their experience of practising assertive communication on both organizational and societal levels. These changes are reflected by the unprecedented transformations that Saudi society is currently going through, and the publication of *Vision 2030* which lays out the blueprint for an ambitious reform, not only in the economic and public service sectors but also in many other sectors such as healthcare and education. A fundamental pillar of this vision is to embrace transparency and accountability reforms and restructure the healthcare system with substantial investment in the healthcare workforce. Two specific subthemes emerged from this theme: The role of hospital accreditation and leadership shift.

#### 4.3.1. The Role of Accreditation

According to many participants, the hospital accreditation process plays a key role in promoting the culture of assertive communication in hospital settings. More specifically, empowering staff to speak up is one priority that the hospital senior management team aims to fulfill to proceed with the successful accreditation process. This has helped in supporting nurses and other healthcare professionals to exercise assertive communication practice and escalate issues related to preventing patient harm:

“… You know, the hospital has been accredited by Joint Commission accreditation and also … Central Board for Accreditation of Healthcare Institutions (CBAHI), and part of their requirements is to know how to escalate any issue, in order to solve it and prevent any harms…”- Director of pharmacy

As a part of the accreditation process, a new set of hospitalwide management and leadership skills had to be implemented. These skills are aimed at empowering all healthcare professionals in the hospital, and inevitably, embedding the practice of assertive communication and speaking up against unsafe practices becomes a priority:

I think assertive communication for our organization has been implemented recently … before people are not assertive in their communication … but when we are improving with the accreditation and the meaning of communication, and how to approach this staff and the other people… … we can now express our (…) ideas and feelings in an open and direct way, and honest way so it's helped us a lot … let's say within five years with the accreditation of Joint Commission International (JCI) and with the CBAHI accreditation, this concept (assertiveness at work setting) become more clear for us and more applicable unlike before. Assistant director of nursing services for training and education

One nurse illustrated how fulfilling the requirements of the hospital accreditation process has led to a greater tendency from doctors to react more constructively to nurses' concerns, although the extent and magnitude of this shift are much less clear.

Moderator … so all of them (doctors) ignoring nurses' views, or some of them?

Female non-Saudi nurse:

It was happening before… all of the doctors…they are trying to listen. Yes, after accreditation, they respond more.

There have been explicit clues that whilst going through national and international hospital accreditation is a major achievement, it has provoked an important discussion on reshaping the safe, effective, and therapeutic communication skills and teamwork in the hospital working culture. The medical director explained how the accreditation process has impacted the multidisciplinary decision-making process in a hospital setting.

They (the accreditation team) have chapters and standards. One of them is actually leadership and governance and through that, it shouldn't be a one-man decision. It should be a board and everybody, the stakeholders, they should come into the decision-making. So, I cannot decide on behalf of the nurse. I cannot decide on behalf of the transportation because … they have to come up and we have to put a policy to be executed by everybody else. So these accreditations opened our eyes that this is the way *we have to shift and improve our culture towards the target*. Medical director

The medical director was eager to not only showcase the conscious efforts to turn the tide and prioritize the culture of empowering hospital staff to exercise assertive communication, but also acknowledged that resisting forces are hindering the progress of these efforts.

We are injecting these concepts (i.e., empowerment) into the practice. You still have part of the organization and culture. I think that's the biggest challenge now, is how to get this culture into the new culture with people and empowering everybody. You have to empower your housekeeper to do stuff rightly, cause that's a reflection on the patient care … So we are injecting that. There are areas of resistance, but at least we are doing that. Medical director

#### 4.3.2. Leadership Shift

There was a sense of leadership shift driven by generational gaps. The generational gap has provided the platform for transforming old work practices and endorsing new ones. The newcomer leaders tend to be young, more energetic, and embrace fresh perspectives that are more closely aligned with empowering the healthcare workforce as advocated by Saudi *Vision 2030*:

“she (new senior member of the nursing team) just joined recently from Australia where she did her research… in the quality of nursing she tried her best to bring awareness to staff on communication and to create a no blame culture environment.” Assistant director of nursing services for training and education

These leaders are leading by example, rather than communicating it verbally, and this sends a powerful message of support to front-line staff, including bedside nurses. This appears to be a critical driver for embedding more assertive communication, particularly when it comes to safeguarding patient safety. One nurse was resolute in showcasing the support he received from the senior management team when it came to expressing views and suggestions.

“My head nurse, my supervisor… the management here all nursing directors. They are, they will be with you …” Male Saudi nurse

Similarly, when genuine support is forthcoming not only from the senior nursing management team but also from those senior hospital leaders who are being challenged by nurses, the impact of empowering those nurses to speak is very conspicuous. One medical director described his reaction when he was once challenged by a nurse from the intensive care unit (ICU):

“… I was once stopped by a junior nurse in the ICU because I had a small cup of coffee and she said, you are not allowed to go, and I say thank you very much. …” Medical director

The shift in the leadership style was also echoed by one pharmacy director, who compared the situation in the past, and how it seems to unfold nowadays:

… some physicians take orders from their seniors and they try to apply them sometimes in the wrong way, and when you try to discuss with them, they said, well, this is what the consultants need, or this is what I've been asked to do. …If anybody has evidence to do such practice, you ask him to present this evidence…. (Now) we ask everybody no matter how, how big you are in a place, it's a learning process. Don't say: I am the godfather of the knowledge and please don't touch me. No, you learn. The first question we ask any practitioner is, do you have evidence? Please present if they have evidence. Pharmacy director

##### 4.3.2.1. Document Analysis

In the documents examined, the concept of speaking up was overall more visible than the phrases of assertive communication skills in those documents examined, but both terms were addressed very implicitly, mainly through the concept of nursing empowerment, which was addressed more conspicuously in the accreditation documents.

A rhetoric that loudly argued for nursing empowerment was evident in several documents examined, including the hospital's annual report and accreditation reports. The nursing competencies and in-house training stressed the fact that nurses must “escalate” any concerns through proper communication channels, and the nurses needed to know the system for doing so, whether it is verbally or via online reporting. Communication protocols/policies published on the intranet illustrated the decision-making process when it comes to escalating patient safety concerns and resolving conflict management. However, there was little operational guidance on how nurses can overcome the challenges of escalating/speaking up or acting assertively in the first place. For example, how to break the power imbalance among junior and senior nurses, and those from other professions, such as doctors. Moreover, there was no illustration of specific procedures or tactics that nurses can adopt to accommodate the challenges associated with expressing assertiveness in the work culture. A clear example can be demonstrated in the nursing preceptorship program, where there was an emphasis on communication skills, but a very implicit focus on what practical steps nurses can utilize to embrace assertive communication. Those two concepts were incorporated into the ethic of escalating concerns, and the prevailing discourse that support will be available if needed. Furthermore, the lack of an operational definition of what counts as “speaking up” behavior may have created an area of ambiguity among the nursing workforce, so when confronting unsafe practice, the nurses may employ communication tactics that are socially untested or culturally inappropriate.

## 5. Discussion

### 5.1. Understanding the Cultural Context

The conspicuous views in this research not only support the dominant cultural conviction that individuals are expected to submit to decisions of those in power, in return for fitting well in the team and the wider community, but also to unlock access to social, professional, and work-related privileges that would otherwise be difficult, if not impossible, to gain access to. Challenging this cultural conviction is seen as the exception (i.e., swimming against the current) and would be perceived as undermining the authority and credibility of those making the decisions. Such discourse puts this working culture firmly into the collectivist end of the individualism–collectivism spectrum [1], because it captures the essence of the collectivist culture, where the need to fit well into the community supersedes other social priorities, such as explicitly disagreeing with the decisions of those holding the power.

It was obvious from the participant's views that getting to know the individuals personally in the healthcare team plays a substantial role in fostering the practice of assertive communication among team members. These personal and social bonds seem to redefine who is able, and indeed allowed, to speak assertively and challenge the views of surrounding colleagues. In the nursing context, this seemed to have provided those nurses with greater confidence to express their views more assertively, particularly if those nurses hold critical skills or expertise which other team members depend on to make their clinical and professional decisions. This finding confirms previous evidence on the impact of social association on the decision-making process for both practising and responding to assertive communication behavior [11], and that establishing a trusting relationship is a prerequisite for expressing assertive views [35], implying that it is often awkward to speak assertively to healthcare professionals whose face is unfamiliar, and come from outside of nurse' social circle, at least in the collectivist culture. From a practical perspective, getting the nurses to know each other and socialize, whether in a formal or nonformal way, is likely to break the barrier among the nursing and other healthcare professional team members. Interprofessional socialization refers to the skills, knowledge, attitudes, and beliefs that inform and affect interprofessional practice behaviors [36]. That can be facilitated partly by involving all healthcare team staff in formal and informal meetings and the decision-making process whenever possible. Previous studies found that improved socialization among interprofessional team members in a clinical environment led to better understanding and appreciation for the roles and responsibilities of other healthcare professionals, enhanced professional confidence, and more effective communication [37].

The views of expat nurses were largely subservient, avoiding direct confrontation whenever possible. Although there is no conclusive evidence as to why they adopt such communicative behavior, job insecurity is one explanation that is frequently cited in expat nursing literature in Saudi Arabia and elsewhere [38, 39]. Individuals tend to reflect on both the risks and benefits of expressing assertive views, as there are bound to be instances when being assertive may not be the most appropriate course of action, such as in situations that may result in the potential for injury to themselves or others [40]. This submissive behavior is not only reinforced by the desire to comply with the social norms and expectations which endorse obedience and discourage disagreement with those in authority [41], but also, most of the expat nurses who were interviewed were either Indian or Philippines, both cultures subscribe to the collectivist paradigm [1]. So, even if they are willing to express their assertive views, their cultural tenets employ further constraints on them to do so.

### 5.2. Tactics for Practicing Assertiveness

Avoiding public showdown is a key strategy utilized by many participants to maintain the delicate balance between two strongly contrasted priorities: Upholding the Saudi sociocultural norms which emphasize submissive attitudes, and expressing an assertive message that clearly conveys disagreement and speech refusal. Bringing such disapproval in front of the public would not only constitute a major departure from the deeply-rooted values of collectivist Saudi culture but also foster a sense of humiliation for the interlocutor [42]. Avoiding a public showdown aims mainly at “saving face” of the individual being challenged. This practice is considered a central factor that underpins the cultural identity of Saudi Arabia, and should therefore be preserved. These findings confirmed previous ones, which suggest that Saudis are eager to “save face,” particularly when engaging in a refusal speech act [16, 43]. Similarly, Vallauri [44] suggested that assertive and refusal messages can be seen as threatening to the interlocutor; therefore, all parties are bound to employ pragmatic strategies to safeguard the face of their interlocutor. In line with this assumption, avoiding public showdown emerged in this study as a key, mutually agreed strategy for unifying all parties on saving the face of both the speaker and the listener.

On many occasions, the nursing participants tend to negotiate the way to communicate assertively, particularly when dealing with those in higher authority (i.e., senior colleagues) or members from perceived higher professional status (i.e. physicians). They adopt certain strategies such as throwing questions, as opposed to directly saying no, which reminds the physician, in a subtle way, of the nurses' refusal act. These strategies have been highlighted in the literature, which again not only reflects the collectivist classification of Saudi culture but also reinforces the importance of face-saving strategies represented in indirect or implicit refusal [16]. The negotiated communication strategy is to say no without saying no [42]. This largely conforms with the previous evidence of the doctor–nurses game [45], which aims at avoiding direct confrontation between nurses and doctors by using implicit, yet mutually agreed strategies to keep healthy working relationships. The nurses' practice in this context not only conforms to the Saudi cultural norms but is further amplified by keeping the traditional way of articulating communication channels in the context of the doctor–nurse game, which portrays the nurses' attempts to indirectly correct doctors or provide input in ways which do not compromise the authority of doctors [46].

### 5.3. Change on the Horizon

#### 5.3.1. Role of Accreditation

On organizational levels in Saudi Arabia, all government and private hospitals are mandated to achieve accreditation by the national accreditation body, such as the CBAHI, or an international one, such as the JCI based in the United States of America. A search to date has found that over 109 hospitals have been accredited by the Joint Commission in Saudi Arabia, showing the ambition that the healthcare sector has made compared to previous years [47]. Navigating a new era of accreditation has brought about standardization of safe practice to enhance the quality of care and to focus on both ends of healthcare, from a patient's perspective to a healthcare professional.

Going through hospital accreditation can be complex and laborious, yet it demonstrates the organizational commitment to empowering nurses, with additional improvement in job commitment, motivation, and employee satisfaction. A study conducted in three hospitals in the Mecca region in Saudi Arabia found a significant positive relationship between nurses' empowerment and their perceived organizational commitment [48]. This commitment includes engaging the nurse employees in all organizational processes and standardizing all clinical policies and procedures.

#### 5.3.2. Leadership Shift

Along with the vast changes dictated by *Vision 2030* (Vision 2030, [49]), young, qualified Saudis are now accepting leadership positions and managerial practices more than before. With these new changes, Saudis are becoming role models early in their careers utilizing transformational leadership as has been documented in Saudi and the wider Gulf region [50, 51]. The participants' views suggest that they are aspiring for elements of transformational leadership, which is guided by *Vision 2030*. Transformational leadership is reported to foster and sustain a positive organizational culture [50]. Leaders need to build a clear vision and philosophy in order to communicate expectations, develop staff, and lead healthcare organizations to achieve strategic goals either on an organizational level or nationally. The way transformative leaders interact with their followers defines both leaders and followers' motivation and integrity [50, 51].

One of the main barriers to effective communication described by nurses in the study was the inner conflict between departments, leading to an impeding communication process. Al-Kalaldeh et al. [52] examined communication barriers among nurses and found that the third most common factor ranked by nurses was work conflicts. However, leading by example, it was one of the key practices that the participants were keen to showcase in this research, and it is one of the successful transformational leaders' components described in the literature [53]. It is the way in which one exemplifies the actions and behaviors which are deemed correct or beneficial for a productive and positive work environment.

### 5.4. Limitations

The participants had varying degrees of English language proficiency, which might have otherwise captured the full complexities of the key issues in understanding communication skills from the participants' perspectives. To mitigate this problem, the participants were allowed sufficient time to express their views, reparaphrase their words, and also renarrate their stories again using different phrases if they wanted to. The expat nurses in the focus group appeared to be cautious when expressing their views, and their answers were mainly short and sharp, although the moderators adopted a few strategies to encourage them to open up (i.e., reassurance about the confidentiality of the views expressed and conducting a focus group with expat nurses only). Their cautious attitudes may have impacted the quality of the data collected from them, potentially concealing many of their important views. Nonetheless, the data were overall rich, and diverse views were expressed in the focus groups. Moreover, expat nurses come from a collectivist culture, so it would be interesting to examine the views of those nurses who come from an individualist paradigm, and whether they accommodate both the current cultural expectations with their own when it comes to practising assertive communication attitudes.

## 6. Conclusion

This study provided an important perspective from nurses and other healthcare professionals on how nurses in Saudi Arabia practice assertive communication, and how this phenomenon is contextualized and received among the nursing workforce in the Saudi culture. The cultural context plays a significant role in shaping the tactical choices of how nurses express and respond to assertive messages. Nurse managers and policy makers should now consider how to create a working culture that not only endorses assertive communication among the nursing workforce but also accommodates the cultural factors that are critical to incorporating and sustaining assertive communication behavior. Embedding assertive communication in the working culture in Saudi Arabia dictates unique practical considerations such as reconsidering alternative communication channels for expressing assertive behavior publicly, which can be interpreted as personal humiliation. Further research is needed to examine how these choices are affected by the sociocultural changes that Saudi Arabia is currently witnessing.

The drive for quality care transformation and setting high expectations for the public in terms of how healthcare is managed and delivered, all mean that change is inevitable. Staff empowerment is a key area that will be targeted to improve care quality and foster a culture of safe, assertive communication. A shift in leadership style and working culture, along with international accreditation of healthcare settings, is likely to drive staff empowerment and may lead to the development of previously unknown tactics for expressing assertive communication practice, including the nursing workforce; however, the actual impact is yet to be seen.

## Figures and Tables

**Table 1:** The demographic characteristics of the participants.

Data collection methods	Participants	Saudi		Non-Saudi		Total
		Male	Female	Male	Female	
Focus-group interviews	• 1^st^ Focus group (nurses)	3	3			6
	• 2^nd^ Focus group (nurses)			3	4	7
	• 3^rd^ Focus group (doctors and pharmacists)	2	1		2	5
	• 4^th^ Focus group (middle nursing managers such as head nurses and supervisors)		5	1		6

Semistructured interviews	• Medical director	1				4
	• Director of pharmacy	1				
	• Director of nursing		1			
	• Assistant director of nursing		1			

**Table 2:** Themes and subthemes.

Major theme	Subthemes
Understanding the cultural context	• Swimming against the current
	• Being acquainted with doctors and nurses
	• The context of expat nurses

Tactical choices for practicing assertiveness	• Avoiding public showdown
	• Aiming for a compromise

Change on the horizon	• Role of accreditation
	• Leadership shift

## Data Availability

The datasets generated and/or analyzed during the current study are available from the corresponding author on reasonable request and are subject to the approval of the relevant Research Ethics Committee.
